# Effectiveness of leniolisib in reducing infections among patients with activated phosphoinositide 3-kinase δ syndrome (APDS)

**DOI:** 10.1007/s12026-026-09863-2

**Published:** 2026-10-24

**Authors:** Niraj C. Patel, Jessica Kuivinen, Sean D. MacKnight, M. Elizabeth M. Younger, Guillaume Germain, Susannah Ripley, François Laliberté, Jesse Fishman, Amanda Harrington

**Affiliations:** 1 Division of Pediatric Allergy and Immunology, Duke University, Durham, NC US; 2 PANTHERx Rare Pharmacy, Pittsburgh, PA US; 3 Groupe d’analyse SRI, Montréal, QC Canada; 4 Division of Allergy, Immunology and Rheumatology, Johns Hopkins University, Baltimore, MD US; 5 Consultant for Pharming Healthcare, Inc, Warren, NJ US; 6 Pharming Healthcare, Inc, Warren, NJ US

**Keywords:** APDS, Leniolisib, Activated phosphoinositide 3-kinase δ syndrome, Infections, Primary immunodeficiency, Real-world evidence

## Abstract

APDS is a rare inborn error of immunity characterized by clinical manifestations, including recurrent infections. This real-world, retrospective analysis evaluated patient-reported infection rates before and after leniolisib initiation. Patients (aged ≥ 12 years) newly initiating leniolisib therapy were eligible for inclusion. Data were retrieved from PANTHERx Rare Pharmacy. Patient-reported infection rates during the baseline period (one year before leniolisib initiation) were compared with infection rates after leniolisib initiation (observation period; from initiation to end of data or death) using generalized estimating equations Poisson regression models with robust standard errors. Patients who had an infection assessment collected via a standardized questionnaire during the baseline and observation periods were included in the analysis (*N* = 71); subgroups for baseline infection rates (lower and higher than the median) and age (12–17 and ≥ 18 years) were also evaluated. The mean (SD) rate of infections per patient-year (PPY) decreased significantly—by 68%—from 5.8 (5.1) during baseline to 1.9 (2.0) in the observation period (rate ratio [RR], 0.32; 95% CI, 0.24–0.42; *P* < 0.001); infections PPY decreased by 38% in patients with low baseline infection rates (RR, 0.62; 95% CI, 0.43–0.89; *P* = 0.010) and 76% in those with high baseline infection rates (RR, 0.24; 95% CI, 0.17–0.34; *P* < 0.001); infections PPY decreased by 70% in those aged 12 − 17 years (RR, 0.30; 95% CI, 0.20–0.45; *P* < 0.001) and by 67% in those aged ≥ 18 years (RR, 0.33; 95% CI, 0.23–0.49; *P* < 0.001). Real-world evidence demonstrated that leniolisib treatment provided a significant reduction in infection rates overall and among subgroups.

## Introduction

Activated phosphoinositide 3-kinase δ syndrome (APDS) is a rare inborn error of immunity characterized by immunodeficiency and immune dysregulation [1–4]. Patients with APDS have either gain-of-function variants in the phosphatidylinositol-4,5-bisphosphate 3-kinase catalytic subunit δ (*PIK3CD*) gene or loss-of-function variants in the phosphoinositide-3-kinase regulatory subunit 1 (*PIK3R1*) gene; these genes encode subunits of the signaling protein phosphoinositide 3-kinase δ (PI3Kδ) [5, 6]. These variants lead to overactivation of PI3Kδ, increased activation of mechanistic target of rapamycin (mTOR) signaling pathways, and persistently compromised immune function that may worsen over time [3].

Clinical manifestations of APDS are highly variable and may include infections within various organ systems, inflammatory or autoimmune disease, malignancy, and neurodevelopmental delay [2]. The median estimated lifespan of patients with APDS is 64 years, 11 years less than the general population, with lymphoma and infections reported as the most frequent causes of death [3, 7]. Recurrent infections are the most common manifestation of APDS and are often a patient’s first presenting symptom [8]. Respiratory infections are the most frequent, but other types—including persistent herpesvirus infections and additional viral or bacterial infections—may also occur [2]. Based on 2023 cost estimates (US dollars), infections have been reported to contribute substantially ($31,711) to the overall mean annual cost per patient with APDS ($116,387), second only to gastrointestinal manifestations ($32,573) [9]. These costs are likely related to frequent emergency department visits and hospitalizations, driven by the severity of these recurrent infections [10].

Leniolisib, the only available targeted treatment for APDS approved by the US Food and Drug Administration, selectively inhibits PI3Kδ to reduce hyperactive signaling [11]. Hyperactive PI3Kδ signaling can alter B- and T-cell development, leading to increased transitional B cells and decreased memory cells—a state that underlies recurrent infections in patients with APDS [1, 12, 13]. Leniolisib was first approved in the United States in March 2023 and is also approved by the European Commission, and in the United Kingdom, Israel, Canada, South Korea, and Australia for the treatment of APDS among individuals aged ≥ 12 years [11, 14–18]. Leniolisib is also approved in Japan for the treatment of APDS in individuals aged ≥ 4 years [19]. During the clinical development program, oral doses of 70-mg leniolisib taken twice daily for 12 weeks significantly reduced lymphadenopathy and increased the percentage of naïve B cells out of total B cells among patients with APDS, suggesting improved immune function and a correction of the underlying immune deficit [20]. In an open-label extension study including patients who had taken leniolisib for > 5 years, patients had a statistically significant reduction in annualized infection rates with each additional year of leniolisib treatment (*P* = 0.0040) as well as a reduction in the number of infection days [21]. Among patients (*n* = 27) who were receiving immunoglobulin replacement therapy (IRT) at the start of the study, 37% had reduced or discontinued IRT use (15% with a reduction and 22% with a discontinuation). Additionally, treatment was well tolerated, and patients experienced sustained reductions in lymphoproliferation.

Despite robust clinical evidence of leniolisib efficacy and tolerability [20–24], the effectiveness of leniolisib outside of a clinical trial setting is not fully understood. This retrospective study examined changes in infection rates before and after leniolisib initiation among patients with APDS.

## Methods

### Study design and participants

This noninterventional, retrospective analysis was conducted using specialty pharmacy data from PANTHERx Rare Pharmacy, the exclusive dispenser of leniolisib in the US. Data were collected between April 5, 2023, and January 9, 2025, and included patients aged ≥ 12 years with APDS who had newly initiated treatment with leniolisib. Patients were ineligible for inclusion if they had prior exposure to leniolisib through participation in a clinical trial or an expanded access program (EAP). After eligible patients were identified and enrolled, demographic and clinical characteristics, as well as the first shipping date of leniolisib for each patient, were retrieved from the PANTHERx Rare Pharmacy’s proprietary specialty workflow technology (SWFT) patient engagement platform.

**Fig. 1:**
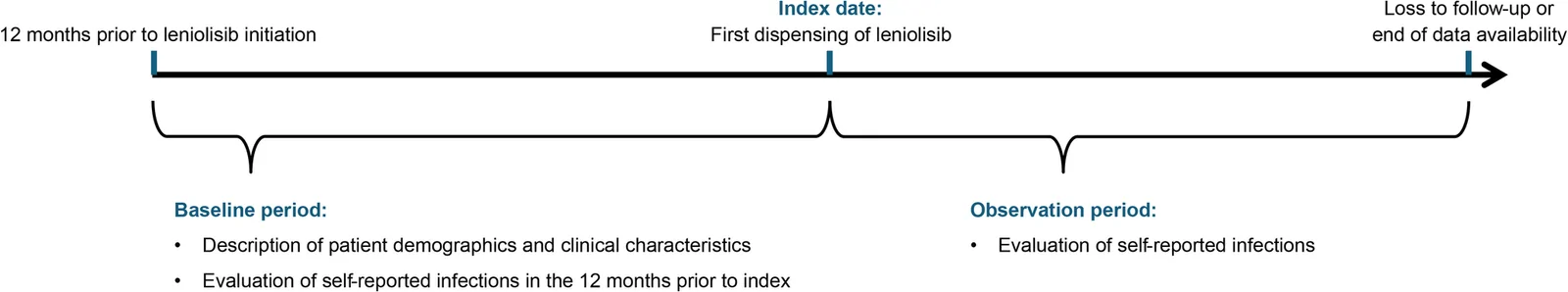
Study design. The first dispensing of leniolisib was the index date. The baseline period included the 12 months preceding the index date, including the index date; the observation period included the time from index date through loss to follow-up (i.e., the date of the patient’s last infection assessment) or end of data availability (January 9, 2025), whichever came first

The first documented shipping date of leniolisib for each patient was defined as the index date. The study was divided into 2 periods (Fig. 1): the baseline period, defined as the 12 months preceding the index date, including the index date, and the observation period, which spanned from the index date to the date of the patient’s last infection assessment or end of data availability (January 9, 2025), whichever came first. Patients who stopped completing infection assessments were considered lost to follow-up. Patients were censored from the infection analysis at each relevant time point (e.g., 6, 9, or 12 months) if the corresponding infection assessment was not completed because the scheduled assessment date occurred after the end of data availability (January 9, 2025).

Each patient was contacted by telephone for an onboarding assessment to document their recalled infection frequency during the year before leniolisib initiation. Pharmacists used a standardized script for onboarding and conducted quarterly calls with patients to collect information, including infection assessments. For the baseline infection assessment, pharmacists asked the patients, “*How many infections would you say you experience each year*?” Patients who needed clarification of what constituted an infection were instructed to include any infection that required treatment. During the observation period, pharmacists systematically collected the number of reported infections from each patient every quarter by asking patients, “*How many infections would you say you have experienced in the last 3 months*?” Patients from the study population were eligible for inclusion in the infection analysis population if they underwent an infection assessment during onboarding and the observation period. Patient-reported infection rates were assessed overall and for the following subgroups: patients with baseline infection rates at or above the median, patients with baseline infection rates below the median, patients aged 12 to 17 years at index, and patients aged ≥ 18 years at index.

The data contained no direct identifiers, and the study was granted exemption by the WCG institutional review board.

### Statistical analysis

Patient-reported infection rates were compared before and after leniolisib initiation using rate ratios (RRs), 95% CIs, and *P* values derived from generalized estimating equations (GEEs) Poisson regression models with robust standard errors. Rates of infection were reported per patient-year (PPY) of observation, which was then weighted by each patient’s observation period to account for varying observation period time. GEE models accounted for within-individual correlation, while the pre-post design allowed each individual to act as their own control, reducing the possibility of confounding. Specifically, an event rate for each patient (number of events divided by the individual observation period time in years) was calculated, and then the overall rate was calculated as the average of these individual rates weighted by each patient’s observation period time in years. This is equivalent to a standard person-time approach, in which the pooled rate equals the total number of events divided by the total observation period time summed across all patients, so that patients with longer observation periods contribute proportionally more to the estimate.

## Results

### Study population

**Fig. 2:**
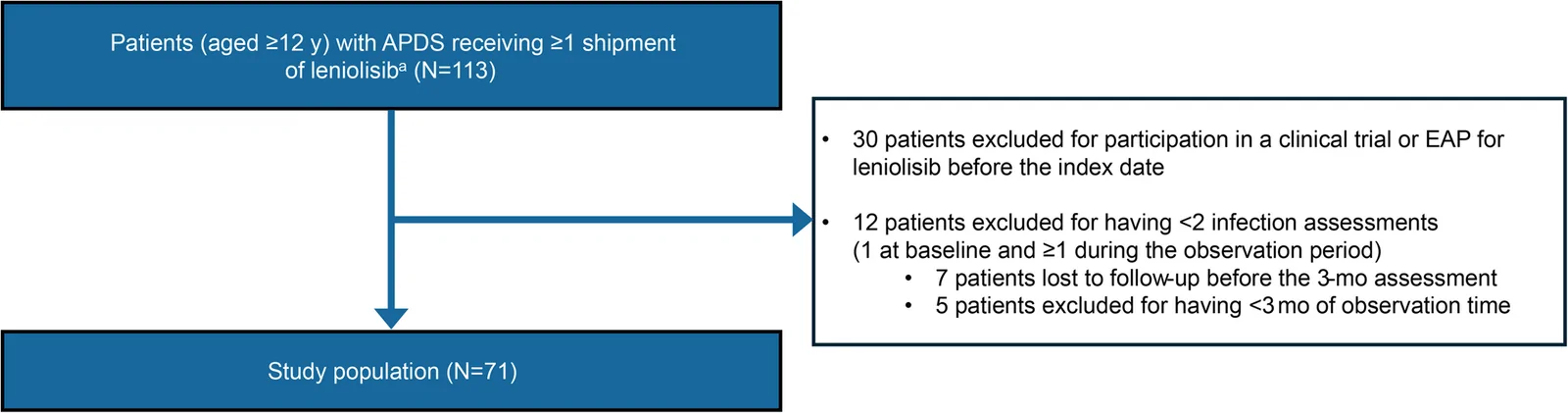
Patient disposition. ^a^The first dispensing of leniolisib was the index date. APDS, activated phosphoinositide 3-kinase δ syndrome; EAP, expanded access program

Of 113 patients with ≥ 1 dispensing for leniolisib, 71 were included in the study population (Fig. 2). Thirty patients were excluded due to previous clinical trial participation or use of an EAP. In the study population, females represented 50.7% of the population, and the mean (SD) age was 25.8 (13.4) years; 42.3% of patients were between 12 and 17 years of age, and 57.7% were ≥ 18 years of age (Table 1). Most patients (80.3%) were prescribed leniolisib by an allergy/immunology specialist, and 54.9% were insured through Medicaid. The mean (SD) length of the observation period was 11.1 (4.2) months, with a median (quartile [Q]1–Q3) of 12 (7–14) months.

**Table 1:** Patient demographics and characteristics

Characteristic	Study population (*N* = 71)
**Age,** ^a^ ** y**	
Mean (SD)	25.8 (13.4)
Median (IQR)	21 (15–35)
**Age group**,^**a**^**n (%)**	
12–17 y	30 (42.3)
≥ 18 y	41 (57.7)
**Gender**,** n (%)**	
Female	36 (50.7)
Male	35 (49.3)
**Prescriber specialty**,** n (%)**	
Allergy/immunology	57 (80.3)
Pediatric	9 (12.7)
Specialist	5 (7.0)
Primary care	4 (5.6)
Hematology/oncology	2 (2.8)
Nurse practitioner	1 (1.4)
Infectious disease	1 (1.4)
Internal medicine	1 (1.4)
**Insurance plan type**,^**b**^**n (%)**	
Medicaid	39 (54.9)
Commercial	27 (38.0)
Medicare	4 (5.6)
Unknown	1 (1.4)

^a^Age at index date

^b^Insurance plan type was categorized according to first dispensing that fit any of the 3 listed insurance types. If a plan type was not listed at first dispensing, the first subsequent dispensing at which the plan was listed was used and reported. Those without any recorded insurance plan type were listed as unknown

A total of 12 patients were excluded due to fewer than 2 infection assessments (1 during the baseline period and ≥ 1 during the observation period). One patient (aged 31.6 years at index; 2 infections reported during the baseline period) died (cause unknown) after 3.0 months of leniolisib treatment and before becoming eligible for an infection assessment in the observation period. The 12 excluded patients were broadly comparable with the study population with respect to age and sex; the mean age of excluded patients was 27.3 years and 58.3% were female. The mean baseline infection rate in the 12 excluded patients was 8.7 infections PPY, largely driven by 2 individuals with high baseline infection rates (20 and 24 infections PPY, respectively). Exclusion of these 2 outliers reduced the mean baseline infection rate to 6 infections PPY among the 10 remaining patients.

Of the 71 patients in the study population, 37 patients had missing infection assessments at 6 (*n* = 12), 9 (*n* = 14), and 12 months (*n* = 11) with mean baseline infections rates of 5.3, 6.9, and 5.1 infections PPY, respectively. Of these, 19 patients were censored because they did not complete the infection assessment at 6 (*n* = 6), 9 (*n* = 7), and 12 months (*n* = 6), as the scheduled assessment dates occurred after the end of data availability. A monotone censoring pattern was observed, such that patients contributed no further observations after loss to follow-up or censoring. One patient (aged 49.3 years at index; 6 infections reported in the baseline period) treated with leniolisib for 13.6 months died before completing the 12-month infection assessment. The reported causes of death were septic shock, lactic acidosis, *Staphylococcus*-related pneumonia, and multiorgan failure.

### Infection rates before and after leniolisib treatment

**Fig. 3:**
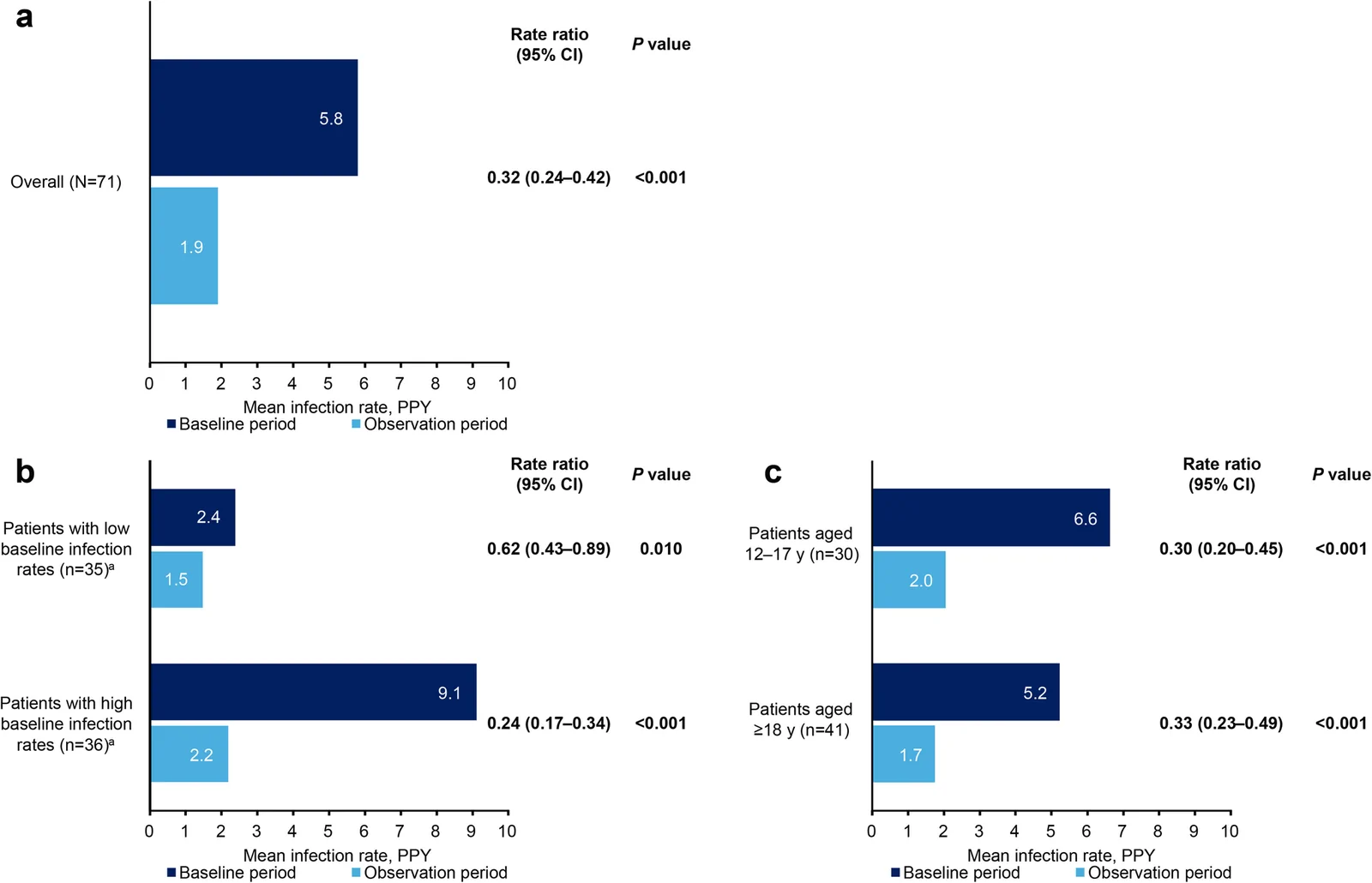
Infection rates during the baseline period and after treatment with leniolisib in **(a)** the study population, **(b)** patients with low and high baseline infection rates, and **(c)** patients aged 12–17 years and those aged ≥ 18 years. ^a^The low infection subgroup comprised patients reporting < 5 infections during the baseline period, and the high infection subgroup comprised those reporting ≥ 5 infections; subgroup classification was based on the overall median rate of baseline infections in the study population. PPY, per patient-year

All 71 patients experienced ≥ 1 infection during the baseline period, and 44 patients (62.0%) experienced ≥ 1 infection during the observation period. The rate of infections PPY decreased significantly by 68% after initiating treatment with leniolisib. The mean (SD; Q1–Q3) infection rate was 5.8 (5.1; 2.0–7.0) infections PPY during the baseline period and 1.9 (2.0; 0.0–2.9) infections PPY during the observation period, corresponding to an RR of 0.32 (95% CI, 0.24–0.42; *P* < 0.001) (Fig. 3).

The median rate of infections during the baseline period was 5, which was used to define patient subgroups: low baseline infection rate, < 5 infections PPY (*n* = 35); high baseline infection rate, ≥ 5 infections PPY (*n* = 36). The mean (SD) length of the observation period was 10.6 (4.6) months, with a median (Q1–Q3) of 12 (7–14) months for the low baseline infection subgroup, and 11.6 (3.8) months, with a median of 13 (10–14) months for the high baseline infection subgroup. Significant reductions in infections were observed in both groups (Fig. 3). Among the 35 patients with baseline infection rates below the median, 19 (54.3%) reported ≥ 1 infection during the observation period; the mean (SD; Q1–Q3) rate of infections PPY decreased from 2.4 (1.2; 1.0–4.0) during the baseline period to 1.5 (1.4; 0.0–2.6) during the observation period, corresponding to a 38% decrease (RR, 0.62; 95% CI, 0.43–0.89; *P* = 0.010). Among the 36 patients with baseline infection rates above the median, 25 (69.4%) reported ≥ 1 infection during the observation period; the mean (SD; Q1–Q3) rate of infections PPY decreased from 9.1 (5.2; 6.0–10.0) during the baseline period to 2.2 (2.4; 0.0–3.5) during the observation period, corresponding to a 76% decrease (RR, 0.24; 95% CI, 0.17–0.34; *P* < 0.001).

In patients aged 12 to 17 years (*n* = 30), the mean (SD) length of the observation period was 11.4 (4.4) months, with a median (Q1–Q3) of 12 (8–14) months; in patients aged ≥ 18 years (*n* = 41), the mean (SD) length of the observation period was 10.9 (4.2) months, with a median (Q1–Q3) of 12 (7–14). Among the 30 patients aged 12 to 17 years, 17 (56.7%) reported ≥ 1 infection during the observation period. The mean (SD; Q1–Q3) number of infections PPY decreased by 70%—from 6.6 (6.0; 2.0–10.0) during the baseline period to 2.0 (2.4; 0.0–3.6) during the observation period (RR, 0.30; 95% CI, 0.20–0.45; *P* < 0.001). Of the 41 patients aged ≥ 18 years, 27 (65.9%) reported ≥ 1 infection during the observation period. The mean (SD; Q1–Q3) number of infections PPY decreased by 67%—from 5.2 (4.2; 3.0–6.0) during the baseline period to 1.7 (1.6; 0.0–2.6) during the observation period (RR 0.33; 95% CI, 0.23–0.49; *P* < 0.001).

## Discussion

These results demonstrate that the robust clinical efficacy of leniolisib for APDS translates into reduced patient-reported infection rates among patients aged ≥ 12 years after initiating leniolisib in a real-world setting [20–24]. Leniolisib was associated with a significant 68% reduction (*P* < 0.001) in patient-reported infections during the 12 months after treatment initiation, decreasing from a mean of 6 infections per year before treatment to 2 infections per year after treatment. This 3-fold reduction may translate to improvements in daily life and to potential cost savings, given that infections comprise approximately 27% of annual medical costs for patients with APDS (mean estimate, $116,387; US dollars, based on 2023 pricing) [9]. Importantly, significant reductions in infection rates were observed in subgroups of patients with baseline infection rates higher (76%; *P* < 0.001) and lower (38%; *P* = 0.010) than the median, indicating that the observed changes were not driven solely by decreases among patients who had the highest baseline infection rates. Furthermore, infection reductions were observed in both adolescents and adults; patients aged 12 to 17 years and those aged ≥ 18 years reported similar significant reductions in infection rates (70% and 67%, respectively; *P* < 0.001 for both).

Although no data were collected regarding infection types, severity, or duration during either the baseline or observation period, the observed decreases in patient-reported infection frequency after initiating leniolisib treatment are consistent with long-term results among patients with APDS who participated in an open-label extension study (OLE) (NCT02859727) [21]. In an interim analysis of 37 patients treated with leniolisib for up to approximately 5 years (median duration of treatment was approximately 2 years) [21], annualized rates of infection decreased significantly by 0.351 (*P* = 0.004) infections with each additional year of leniolisib treatment; there was no appreciable increase in antibiotic use. Among 6 patients followed for up to 6 years [22], 4 experienced a decrease in the number or severity of infections after year 2. Three patients contracted SARS-CoV-2, all of whom experienced mild symptoms. Changes in the infection burden were accompanied by a 2.47% decrease in elevated transitional B cells and increased CD4:CD8 T-cell ratios from 0.63 to 1.11, indicating improved immune regulation. Another study comparing the effects of leniolisib on the annual rate of respiratory infections using data from the OLE study (treatment population) and the European Society for Immunodeficiencies registry (the control population) observed significant reductions in the annualized rate of respiratory tract infections (RR, 0.34; 95% CI, 0.19–0.59) in patients treated with leniolisib compared with those who received standard of care [24]. Immunoglobulin M levels were also significantly reduced (-1.09 g/L; 95% CI, -1.78 to -0.39; *P* = 0.002) in patients treated with leniolisib compared with those who received standard of care, suggesting improved immune function.

A reduction in infection rates may contribute to a reduction in the overall healthcare resource utilization (HRU) observed in patients with APDS, potentially through reduced healthcare-related visits and overall healthcare costs. Retrospective studies before leniolisib approval in 2023 have observed IRT use in 59% to 89% of patients with APDS [2, 10, 25]. Additionally, these studies observed that patients received other treatments that could be used in infection management, including antibiotics (61%–86%), corticosteroids (79%), and antifungals (6%–31%) [2, 10, 25]. In the leniolisib OLE study, treatment with leniolisib reduced IRT use in 37% (10 of 27) of patients receiving IRT at study entry, including reductions in use in 15% of patients and discontinuation of use in 22% of patients [21]. The effects of leniolisib treatment on immune dysfunction and dysregulation and its impact on HRU among patients with APDS will need to be further evaluated outside of the clinical trial setting.

While the present study focused on patients who were ≥ 12 years of age, future studies should determine long-term real-world effectiveness of leniolisib treatment for patients with APDS aged < 12 years. Although the observed age at APDS diagnosis reported in the literature is 12 to 15 years (median [IQR] 12.0 [6.5–21.5], mean [range] 13.4 [0–56], and median [IQR] 15.5 [2–54] years), infections can begin in infants < 1 year of age, and the age of symptom onset is approximately 2 to 4 years (mean [range] 2.0 [0.003–22] years and median [IQR] 4.4 [0.25–28] years) [1, 3, 26]. This diagnostic delay from symptom onset to APDS diagnosis is estimated to be a median (IQR) of 7 (3.4–14.0) years and a mean (range) of 11 (0–44) years [1, 3]. In a systematic review of 351 unique patients, infections were the second most common cause of death (9 patients [22%]); all patients were < 15 years of age, highlighting the unmet need among this population [3].

A limitation of the study is that the standardized pharmacy assessments did not capture microbiologic confirmation, infection etiology, or criteria for distinguishing separate or concurrent infection episodes. Consequently, the outcome reflects patient-reported infection burden rather than clinically adjudicated infection events. Another limitation is that baseline infection data were based on patient recall of infections during the preceding year, which may have introduced inaccuracies in the assessment of baseline infection rates. However, the use of quarterly interviews during the observation period was intended to optimize reporting accuracy. The consistency between these self-reported findings and the results of previous clinical studies provides supportive context for the present observations [2, 21]. Although the pre-post study design allowed patients to serve as their own controls, the potential for time-varying confounding remains. Confounding due to concomitant IRT, corticosteroid use, antimicrobial use, or other changes in clinical management cannot be excluded. Importantly, pre-post study designs are a well-established approach in rare disease research, particularly in settings where placebo-controlled studies may be impractical or infeasible [27]. Moreover, all patients initiated leniolisib during a relatively narrow time frame (2023–2024), outside the COVID-19 pandemic period, reducing the likelihood that substantial temporal changes in infection risk influenced the findings.

A total of 12 patients were excluded from the study population because they failed to complete the 3-month follow-up assessment, raising the possibility of selection bias. One patient who died before completing the 3-month assessment reported 2 infections in the baseline period, lower than the mean of the study population (5.8 infections PPY) and similar to that of the low baseline infection subgroup infection rate (mean 2.4 infections PPY). The 12 excluded patients were broadly similar to the study population with respect to age and sex, and their mean baseline infection rate was comparable to that of the high baseline infection rate subgroup (mean 8.7 vs. 9.1 infections PPY, respectively). After the exclusion of 2 outliers who discontinued treatment, the mean baseline infection rate of the remaining 10 patients was similar to the study population (6.0 vs. 5.8 infections PPY, respectively). Therefore, selection bias is not expected to account for the observed results.

Finally, because the observation period ended at each patient’s last infection assessment, the analysis may have been susceptible to informative loss to follow-up. To account for differences in observation period duration, infection rates were annualized as PPY and weighted according to each patient’s observation time. Although this approach appropriately accommodates unequal observation period durations, it does not address the censoring mechanism itself and therefore cannot eliminate the possibility of informative censoring. Patients with a greater baseline infection burden may have been more likely to discontinue during the observation period because of worsening health, severe infections, death, or other factors and therefore may have contributed fewer patient-years of observation, potentially affecting the estimated infection rates. A total of 37 patients were excluded from the analysis due to missing infections assessments at 6, 9, or 12 months. The baseline infections rates of these patients were similar to the study population (5.3, 6.9, and 5.1 infections PPY, respectively vs. 5.8 infections PPY). Furthermore, in our study, 1 patient died during the observation period; the baseline infection rate for this patient was similar to that of the study population (6 vs. 5.8 infections PPY, respectively). Therefore, loss to follow-up due to early mortality is unlikely to have substantially contributed to the observed reduction in infection rates.

Reassuringly, patients with baseline infection rates above and below the population median (5 infections PPY) had similar observation periods, providing no evidence of substantial differential observation period duration according to baseline infection burden. Moreover, after evaluating the impact of missing observation period infection assessments, we found that mean patient ages and baseline infection rates did not substantially differ across the observation time period or from the study population. Therefore, loss to follow-up is more likely to be non-informative in this case.

## Conclusion

This study provides initial real-world observations of infection rates before and after initiation of leniolisib among patients with APDS. In this retrospective analysis, patients (aged ≥ 12 years) reported experiencing 68% fewer infections per year during treatment with leniolisib compared with the year before initiating treatment. Significant reductions in infections were observed regardless of whether patients had high or low baseline rates of infection and regardless of patient age. Future studies are needed to evaluate the long-term effects of immune reconstitution with leniolisib on infection rates and mortality risks.

## Data Availability

Data are provided within the manuscript. The patient-level data in this study were obtained from PANTHERx Rare Pharmacy and are not publicly available. Further inquiries can be directed to the corresponding author.

## References

[1] Jamee M, Moniri S, Zaki-Dizaji M, et al. Clinical, immunological, and genetic features in patients with activated PI3Kdelta syndrome (APDS): a systematic review. Clin Rev Allergy Immunol. 2020;59(3):323–33. 10.1007/s12016-019-08738-9. 31111319

[2] Coulter TI, Chandra A, Bacon CM, et al. Clinical spectrum and features of activated phosphoinositide 3-kinase delta syndrome: a large patient cohort study. J Allergy Clin Immunol. 2017;139(2):597–e606594. 10.1016/j.jaci.2016.06.021. PMC5292996 27555459

[3] Büsch K, Memmott HL, McLaughlin HM, Upton JEM, Harrington A. Genetic etiologies and outcomes in malignancy and mortality in activated phosphoinositide 3-kinase delta syndrome: a systematic review. Adv Ther. 2025;42(2):752–71. 10.1007/s12325-024-03066-7. PMC11787279 39636570

[4] Poli MC, Aksentijevich I, Bousfiha AA, et al. Human inborn errors of immunity: 2024 update on the classification from the International Union of Immunological Societies Expert Committee. J Hum Immun. 2025;1(1):e20250003. 10.70962/jhi.20250003. PMC12829761 41608114

[5] Angulo I, Vadas O, Garçon F, et al. Phosphoinositide 3-kinase delta gene mutation predisposes to respiratory infection and airway damage. Science. 2013;342(6160):866–71. 10.1126/science.1243292. PMC3930011 24136356

[6] Lucas CL, Zhang Y, Venida A, et al. Heterozygous splice mutation in PIK3R1 causes human immunodeficiency with lymphoproliferation due to dominant activation of PI3K. J Exp Med. 2014;211(13):2537–47. 10.1084/jem.20141759. PMC4267241 25488983

[7] Mahendran M, Upton JEM, Ramasubramanian R, et al. Overall survival among patients with activated phosphoinositide 3-kinase delta syndrome (APDS). Orphanet J Rare Dis. 2025;20(1):212. 10.1186/s13023-025-03734-z. PMC12049806 40319290

[8] Activated phosphoinositide 3-kinase delta syndrome (APDS). National organization for rare disorders. Updated April 4. 2023. Accessed January 6, 2026. https://rarediseases.org/rare-diseases/activated-phosphoinositide-3-kinase-delta-syndrome-apds/.

[9] Hartog NL, Wu EY, Rider NL, et al. Estimated annual direct medical costs of manifestations among patients with activated phosphoinositide 3-kinase delta syndrome. Clin Exp Med. 2025;25(1):246. 10.1007/s10238-025-01773-1. PMC12255542 40652158

[10] Rider NL, Laliberté F, Germain G, et al. Real-world health care resource utilization and costs among patients with activated phosphoinositide 3-kinase delta (PI3Kδ) syndrome in the United States. Adv Ther. 2025;42(12):6059–77. 10.1007/s12325-025-03377-3. PMC12618449 41060524

[11] Joenja. Prescribing information. Pharming Healthcare, Inc.; 2023. https://www.accessdata.fda.gov/drugsatfda_docs/label/2023/217759s000lbl.pdf. Accessed September 1, 2026.

[12] Lucas CL, Kuehn HS, Zhao F, et al. Dominant-activating germline mutations in the gene encoding the PI(3)K catalytic subunit p110δ result in T cell senescence and human immunodeficiency. Nat Immunol. 2014;15(1):88–97. 10.1038/ni.2771. PMC4209962 24165795

[13] Cant AJ, Chandra A, Munro E, Rao VK, Lucas CL. PI3Kδ pathway dysregulation and unique features of its inhibition by leniolisib in activated PI3Kδ syndrome and beyond. J Allergy Clin Immunol Pract. 2024;12(1):69–78. 10.1016/j.jaip.2023.09.016. PMC10872751 37777067

[14] Pharming Group NV. Pharming announces US FDA approval of Joenja (leniolisib) as the first and only treatment indicated for APDS. News release [internet]. March 24, 2023. Accessed November 10, 2025. https://www.pharming.com/news/pharming-announces-us-fda-approval-joenja-leniolisib-first-and-only-treatment-indicated-apds.

[15] Medicines and Healthcare Products Regulatory Agency. MHRA approves first medicine for rare immune disease APDS. News release [internet]. September 27. 2024. Accessed November 10, 2025. https://www.gov.uk/government/news/mhra-approves-first-medicine-for-rare-immune-disease-apds.

[16] Pharming Group NV. Pharming Group provides update on ongoing regulatory review of leniolisib for the treatment of APDS in the European Union. News release [internet]. May 30, 2024. Accessed November 10, 2025. https://www.pharming.com/news/pharming-group-provides-update-ongoing-regulatory-review-leniolisib-treatment-apds-european-union.

[17] Australian Government Department of Health, Disability and Ageing, Therapeutic Goods Administration. Australian public assessment report for JOENJA. June 2025. Accessed November 10. 2025. https://www.tga.gov.au/sites/default/files/2025-07/auspar-joenja-250711.pdf.

[18] Pharming reports second quarter and first half 2026 financial results and revises year-end guidance; strong Joenja momentum and near-term clinical readouts to support broader therapeutic use. News release [internet]. July 30. 2026. Accessed September 4, 2026. https://www.pharming.com/our-news/pharming-reports-second-quarter-and-first-half-2026-financial-results.

[19] Pharming Group announces approval of Joenja^®^ (leniolisib) in Japan for the treatment of APDS in patients aged 4 years and older. News release [internet]. March 24. 2026. Accessed August 25, 2026. https://www.pharming.com/our-news/pharming-group-announces-approval-of-joenja-leniolisib-in-japan-for-the-treatment-of-apds-in-patients-aged-4-y.

[20] Rao VK, Webster S, Šedivá A, et al. A randomized, placebo-controlled phase 3 trial of the PI3Kdelta inhibitor leniolisib for activated PI3Kdelta syndrome. Blood. 2023;141(9):971–83. 10.1182/blood.2022018546. PMC10163280 36399712

[21] Rao VK, Kulm E, Šedivá A, et al. Interim analysis: open-label extension study of leniolisib for patients with APDS. J Allergy Clin Immunol. 2024;153(1):265–e274269. 10.1016/j.jaci.2023.09.032. PMC10841669 37797893

[22] Rao VK, Kulm E, Grossman J, et al. Long-term treatment with selective PI3Kdelta inhibitor leniolisib in adults with activated PI3Kdelta syndrome. Blood Adv. 2024;8(12):3092–108. 10.1182/bloodadvances.2023011000. PMC11222951 38593221

[23] Rao VK, Šedivá A, Dalm VASH, et al. A randomised, placebo-controlled, phase III trial of leniolisib in activated phosphoinositide 3-kinase delta (PI3Kdelta) syndrome (APDS): adolescent and adult subgroup analysis. Clin Immunol. 2025;270:110400. 10.1016/j.clim.2024.110400. PMC11631659 39561927

[24] Whalen J, Chandra A, Kracker S, et al. Comparative efficacy of leniolisib (CDZ173) versus standard of care on rates of respiratory tract infection and serum immunoglobulin M (IgM) levels among individuals with activated phosphoinositide 3-kinase delta (PI3Kdelta) syndrome (APDS): an externally controlled study. Clin Exp Immunol. 2025;219(1):uxae107. 10.1093/cei/uxae107. PMC11754865 39673396

[25] Elkaim E, Neven B, Bruneau J, et al. Clinical and immunologic phenotype associated with activated phosphoinositide 3-kinase δ syndrome 2: a cohort study. J Allergy Clin Immunol. 2016;138(1):210–e218219. 10.1016/j.jaci.2016.03.022. 27221134

[26] Barzaghi F, Moratti M, Panza G, et al. Report of the Italian cohort with activated phosphoinositide 3-kinase delta syndrome in the target therapy era. J Clin Immunol. 2024;45(1):58. 10.1007/s10875-024-01835-1. PMC11666751 39714594

[27] Videnovic A, Pfeiffer HCV, Tylki-Szymańska A, et al. Study design challenges and strategies in clinical trials for rare diseases: lessons learned from pantothenate kinase-associated neurodegeneration. Front Neurol. 2023;14:1098454. 10.3389/fneur.2023.1098454. PMC10032345 36970548

